# SARS-CoV-2 infection and COVID-19 vaccination and the risk for new-onset type 1 diabetes: a register-based population study in Sweden

**DOI:** 10.1007/s00125-026-06767-6

**Published:** 2026-06-06

**Authors:** Huiqi Li, Lisa Morris, Maria Bygdell, Ailiana Santosa, Elin Allansson Kjölhede, Katarina Eeg-Olofsson, Fredrik Nyberg, Yiyi Xu

**Affiliations:** 1 School of Public Health and Community Medicine, Institute of Medicine, Sahlgrenska Academy, University of Gothenburg, Gothenburg, Sweden; 2 Unit of Clinical Pharmacology, Department of Pharmaceuticals, Sahlgrenska University Hospital, Region Västra Götaland, Gothenburg, Sweden; 3 Department of Internal Medicine and Clinical Nutrition, Institute of Medicine, Sahlgrenska Academy, University of Gothenburg, Gothenburg, Sweden; 4 Department of Medicine, Sahlgrenska University Hospital, Gothenburg, Sweden; 5 Department of Molecular and Clinical Medicine, Institute of Medicine, Sahlgrenska Academy, University of Gothenburg, Gothenburg, Sweden; 6 Centre of Registers Västra Götaland, Gothenburg, Sweden

**Keywords:** Cohort, Register study, SARS-CoV-2 infection, Type 1 diabetes, Vaccination

## Abstract

**Aims/hypothesis:**

Reports from several countries suggested increased incidence of type 1 diabetes during the COVID-19 pandemic, but causality has remained unclear. We investigated incident type 1 diabetes related to SARS-CoV-2 infection and vaccination in Swedish children and adults and assessed whether vaccination modified infection-related risk.

**Methods:**

We assembled a register-based cohort of all residents aged <80 years on 1 January 2020 and births during follow-up (1 January 2020–31 December 2023). Risk windows after infection (and after each vaccine dose) were 0–30 days, 31–180 days, 181–365 days, and 1–2 years. Incident type 1 diabetes was defined by the earliest ICD-10 diagnosis E10 in the National Diabetes Register or in the National Patient Register. Cox regression with calendar time as the timescale was used with time-varying exposures; analyses were stratified by age (children <18 years; adults 18–79 years), with age-appropriate covariate adjustment. Sensitivity analyses for children were restricted to ages 12–17 years.

**Results:**

The cohort included 2,650,492 children (3813 incident type 1 diabetes) and 6,870,328 adults (4453 incident type 1 diabetes). SARS-CoV-2 infection was associated with increased type 1 diabetes risk within 2 years in children (hazard ratio [HR] 1.22; 95% confidence interval 1.10, 1.36) and adults (1.10; 1.00, 1.20), driven largely by the 0–30-day window (5.41; 4.34, 6.74 in children, 3.33; 2.69, 4.12 in adults). Vaccination did not modify infection-associated risk (interaction *p*>0.5). When vaccination was investigated as the exposure, children showed lower HRs (0.77; 0.67, 0.88) within 2 years, but this association was not observed in ages 12–17 (1.00; 0.80, 1.26); adults showed a small excess risk within 0–30 days after dose 1 (1.32; 1.07, 1.62), but not in later windows or doses.

**Conclusions/interpretation:**

Short-term elevations in incident type 1 diabetes diagnoses were observed in the first 30 days after infection and after first vaccination, and did not persist, supporting detection/diagnostic acceleration rather than a sustained causal effect of SARS-CoV-2 infection or vaccination on type 1 diabetes.

**Graphical Abstract:**

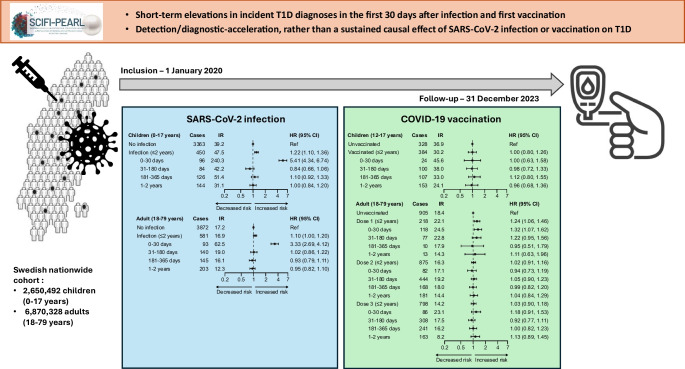

**Supplementary Information:**

The online version of this article (10.1007/s00125-026-06767-6) contains peer-reviewed but unedited supplementary material.


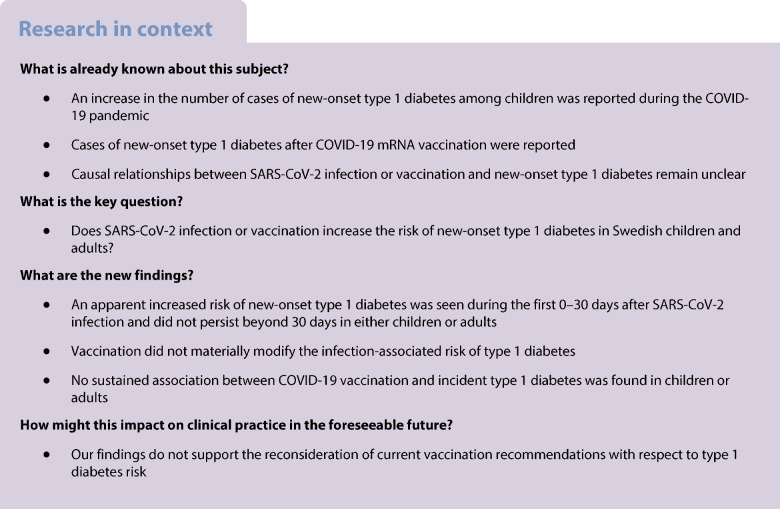


## Introduction

Several observational studies have reported an increased incidence of type 1 diabetes during the COVID-19 pandemic [1–3], especially during the first 2 years [4]. A similar trend was also observed in Sweden, especially among younger children [5, 6]. The rise of type 1 diabetes is deeply concerning as it decreases children’s quality of life and increases the burden on healthcare services [7], which has motivated studies to investigate the possibility of a causal relationship between SARS-CoV-2 infection and type 1 diabetes. However, available studies have yielded inconsistent results, and it is still unclear whether such an increase is directly linked to the infection or changes in other risk factors during the pandemic [6, 8–11].

Another important aspect worth investigating is COVID-19 vaccination. A study from Scotland showed that the sharp increase in type 1 diabetes incidence in 2021 among children aged 6–14 years returned towards pre-pandemic levels by 2022 [12]. COVID-19 vaccines, which were introduced in 2022 to children in the UK, could be one possible explanation. As vaccination in the initial pandemic phase showed acceptable effectiveness against SARS-CoV-2 infection [13], it is reasonable to assume that vaccination could reduce the risk of new-onset type 1 diabetes through the protection against infection. However, there are also case reports of new-onset type 1 diabetes after COVID-19 mRNA vaccination [14], especially in some individuals with a genetic predisposition [15, 16]. In a recent population-based ecological study, childhood COVID-19 vaccination rates were not significantly associated with type 1 diabetes incidence over the subsequent 12 months, indicating no effect of vaccination on type 1 diabetes [17]. These contradictory findings highlight the need to appropriately investigate the role of vaccination in the context of type 1 diabetes during the pandemic.

Additionally, despite the number of studies on type 1 diabetes among children and adolescents, studies on adults are sparse. Only a few case reports on type 1 diabetes in relation to COVID-19 are available and these are limited to a specific subtype of type 1 diabetes [18, 19]. Even though type 1 diabetes is generally considered as a disease with onset at young age, similar incidence rates (IRs) among individuals aged 0–19 years and among individuals 40–100 years of age have been reported in southeastern Sweden [20]. Adults with new onset of type 1 diabetes should not be a neglected population, and understanding how SARS-CoV-2 infection and vaccination impact this population is therefore important.

This study aimed to investigate whether SARS-CoV-2 infection can increase the risk of new-onset type 1 diabetes in children and in adults, and if vaccination can alter such risk. Further, the study aimed to assess if COVID-19 vaccination per se could alter the risk of new-onset type 1 diabetes.

## Methods

### Data sources

This study is part of the RECOVAC (register-based large-scale national population study to monitor COVID-19 vaccination effectiveness and safety) study effort within the larger SCIFI-PEARL (Swedish COVID-19 Investigation for Future Insights—a Population Epidemiology Approach using Register Linkage) project [21], with register data linkage currently extended to cover the whole Swedish population. In this study, we used sociodemographic data from Statistics Sweden, including linkage of children to parents via the Multigenerational Register [22]; cause of death data from the National Cause of Death Register [23]; type 1 diabetes diagnoses from the National Diabetes Register (NDR), which covers more than 90% of individuals with type 1 diabetes in Sweden [24]; and from the National Patient Register (NPR), which includes specialist outpatient visits and inpatient care [25]; comorbidity information from the NPR; SARS-CoV-2 infection data (positive tests) from the National Register of notifiable communicable diseases (SmiNet [26]); and vaccination data from the National Vaccination Register (NVR [27]). SmiNet and NVR are managed by the Public Health Agency of Sweden.

### Study design, population and study period

The study cohort included all individuals who were <80 years old and residing in Sweden on 1 January 2020 as well as newborn babies during the study period (1 January 2020 to 31 December 2023). Anyone who had prevalent type 1 diabetes or type 2 diabetes between 1 January 2015 to 1 January 2020 were excluded, therefore the cohort consisted of individuals without diabetes at the study start. Individuals ≥80 years old were excluded due to relatively lower prevalence of type 1 diabetes and much higher prevalence of other diseases than in the general population. We used age 18 years on 1 January 2020 (the study start) as the cutoff between children (<18 years) analyses and adults (18–79 years) analyses.

The study obtained ethics approval from the Swedish Ethical Review Authority (2020-01800 with amendments). Individual consent to participate is not applicable because this study is register based.

### Exposures: infection, vaccination and risk windows

For investigating the association between infection and risk of new-onset type 1 diabetes, the exposure was the first SARS-CoV-2 infection, identified by the earliest record of a positive PCR test or receiving a healthcare diagnosis (U07.1/U07.2) as indicated by the International Classification of Diseases, 10th revision (ICD-10) from the register sources mentioned above. Four mutually exclusive risk windows after infection were defined: 0–30 days, 31–180 days, 181 days–1 year, and 1–2 years, according to literature review [28–30].

For investigating the effects of vaccination, the exposure was COVID-19 vaccination up to 3 doses (unvaccinated, dose 1, dose 2, and dose 3). The same risk windows as above (0–30 days, 31–180 days, 181 days–1 year and 1–2 years) after each dose were applied. However, for analysis among children, the exposure was only two categories (unvaccinated, vaccinated) as few Swedish children were vaccinated with several doses.

### Outcome

The outcome was new-onset type 1 diabetes (ICD-10: E10), using both primary and secondary diagnosis. The type 1 diabetes diagnosis comes from NDR (covers primary healthcare contacts) and NPR (covers both specialist visits or hospitalisations), and the earliest record from either register was considered as onset date.

### Covariates

We used different sets of covariates for children and adults. For children, we included age, gender (from register data), birth country, parents’ birth country, parents’ socioeconomic status (SES; income, education, occupation), family history of type 1 diabetes (at least one parent with a type 1 diabetes diagnosis in the NPR/NDR), baseline comorbidities (asthma, Down syndrome, obesity), variants of concern (VOC) period when particular virus variants were dominating as the first infection occurred (preAlpha [1 January 2020 to 31 January 2021], Alpha [1 February 2021 to 30 Jun 2021], Delta [1 July 2021 to 31 December 2021] and Omicron [from 1 January 2022] [31]), and vaccination status (unvaccinated and vaccinated). For adults, we used their own SES, included a broader set of comorbidities (cardiovascular disease, hypertension, chronic lung diseases, asthma, chronic kidney diseases, autoimmune diseases, dementia, psychiatric conditions, obesity and cancer), and applied a more detailed vaccination variable (unvaccinated, 1–2 doses, or 3 and more doses). For infection analyses, VOC period was excluded from the covariates set due to its high correlation with infection. Similarly, for vaccination analyses, vaccination status was removed as a covariate to avoid collinearity with the exposure variable, noting that the two were categorised differently.

All covariates were defined at baseline (i.e. pre-pandemic) and remained fixed during follow-up, except VOC period at first infection and vaccination status. Baseline comorbidities were defined using medical history (primary or secondary diagnoses from in- or outpatient specialist care) from the NPR during the 5 years before 1 Jan 2020, based on the relevant ICD codes (electronic supplementary material [ESM] Table 1). VOC period at first infection and vaccination status were treated as time-varying covariates. For instance, a person’s vaccination status could change from unvaccinated to maximum 2 doses on the date of dose 1, and then to 3 and more doses on the date of dose 3. Detailed categorisations of all covariates are provided in Table 1 (children population) and Table 2 (adult population).

**Table 1:** Baseline demographics and medical history in children (0–17 years) resident in Sweden on 1 January 2020 and newborns during the study period from 1 January 2020 to 31 December 2023, overall and by incident type 1 diabetes at the end of follow-up

Characteristic	Type 1 diabetes at the end of follow-up			
	No	Yes	Total	*p* value
*N*	2,646,679 (99.9)	3813 (0.1)	2,650,492 (100.0)	
Age				
0–11 years	1,858,484 (70.2)	2775 (72.8)	1,861,259 (70.2)	<0.001
12–17 years	788,195 (29.8)	1038 (27.2)	789,233 (29.8)	
Gender				
Boys	1,361,978 (51.5)	2184 (57.3)	1,364,162 (51.5)	<0.001
Girls	1 284 701 (48.5)	1629 (42.7)	1,286,330 (48.5)	
Birth country				
Sweden	2,454,901 (92.8)	3651 (95.8)	2,458,552 (92.8)	<0.001
Outside Sweden	191,778 (7.2)	162 (4.2)	191,940 (7.2)	
Parents’ birth country				
Both from Sweden	1,637,901 (61.9)	2861 (75.0)	1,640,762 (61.9)	<0.001
Either from Sweden	370,332 (14.0)	411 (10.8)	370,743 (14.0)	
None from Sweden	602,276 (22.8)	530 (13.9)	602,806 (22.7)	
Unknown	36,170 (1.4)	11 (0.3)	36,181 (1.4)	
Family disposable income				
Low (1st quartile)	647,650 (24.5)	821 (21.5)	648,471 (24.5)	<0.001
Medium low (2nd quartile)	772,704 (29.2)	1221 (32.0)	773,925 (29.2)	
Medium high (3rd quartile)	720,648 (27.2)	1086 (28.5)	721,734 (27.2)	
High (4th quartile)	487,655 (18.4)	676 (17.7)	488,331 (18.4)	
Unknown	18,022 (0.7)	9 (0.2)	18,031 (0.7)	
Mother’s education				
Primary (<9 years)	264,456 (10.0)	318 (8.3)	264,774 (10.0)	<0.001
Secondary (9–12 years)	899,404 (34.0)	1455 (38.2)	900,859 (34.0)	
Tertiary (>12 years)	1,370,944 (51.8)	1975 (51.8)	1,372,919 (51.8)	
Unknown	111,875 (4.2)	65 (1.7)	111,940 (4.2)	
Father’s education				
Primary (<9 years)	305,516 (11.5)	408 (10.7)	305,924 (11.5)	<0.001
Secondary (9–12 years)	1,129,395 (42.7)	1792 (47.0)	1,131,187 (42.7)	
Tertiary (>12 years)	1,031,574 (39.0)	1452 (38.1)	1,033,026 (39.0)	
Unknown	180,194 (6.8)	161 (4.2)	180,355 (6.8)	
Mother’s occupation				
Healthcare worker	388,358 (14.7)	588 (15.4)	388,946 (14.7)	<0.001
Other essential worker	566,684 (21.4)	869 (22.8)	567,553 (21.4)	
Not essential worker	1,360,279 (51.4)	2019 (53.0)	1,362,298 (51.4)	
Unemployed	249,593 (9.4)	305 (8.0)	249,898 (9.4)	
Unknown	81,765 (3.1)	32 (0.8)	81,797 (3.1)	
Father’s occupation				
Healthcare worker	79,096 (3.0)	114 (3.0)	79,210 (3.0)	<0.001
Other essential worker	281,057 (10.6)	376 (9.9)	281,433 (10.6)	
Not essential worker	2,004,072 (75.7)	3034 (79.6)	2,007,106 (75.7)	
Unemployed	128,502 (4.9)	159 (4.2)	128,661 (4.9)	
Unknown	153,952 (5.8)	130 (3.4)	154,082 (5.8)	
Family history of type 1 diabetes				
No	2,610,856 (98.6)	3432 (90.0)	2,614,288 (98.6)	<0.001
Yes	35,823 (1.4)	381 (10.0)	36,204 (1.4)	
Asthma				
No	2,504,077 (94.6)	3549 (93.1)	2,507,626 (94.6)	<0.001
Yes	142,602 (5.4)	264 (6.9)	142,866 (5.4)	
Down syndrome				
No	2,644,291 (99.9)	3801 (99.7)	2,648,092 (99.9)	<0.001
Yes	2,388 (0.1)	12 (0.3)	2400 (0.1)	
Obesity				
No	2,617,208 (98.9)	3753 (98.4)	2,620,961 (98.9)	0.007
Yes	29,471 (1.1)	60 (1.6)	29,531 (1.1)	
VOC periods at first infection				
No infection	2,160,190 (81.6)	3361 (88.1)	2,163,551 (81.6)	<0.001
PreAlpha (1 January 2020 to 31 January 2021)	64,771 (2.4)	80 (2.1)	64,851 (2.4)	
Alpha (1 February 2021 to 30 June 2021)	105, 011 (4.0)	101 (2.6)	105,112 (4.0)	
Delta (1 July 2021 to 31 December 2021)	70,124 (2.6)	61 (1.6)	70,185 (2.6)	
Omicron (from 1 January 2022)	246,583 (9.3)	210 (5.5)	246,793 (9.3)	
Vaccination				
Unvaccinated	1,825,157 (69.0)	3265 (85.6)	1,828,422 (69.0)	<0.001
Vaccinated	821,522 (31.0)	548 (14.4)	822,070 (31.0)	

Values are presented as *n* (%)

*p* values are obtained from *χ*^2^ test

**Table 2:** Baseline demographics and medical history in adults (18–79 years) resident in Sweden on 1 January 2020, overall and by incident type 1 diabetes at the end of follow-up

Characteristic	Type 1 diabetes at the end of follow-up			
	No	Yes	Total	*p* values
*N*	6,865,875 (99.9)	4453 (0.1)	6,870,328 (100.0)	
Age				
18–29 years	1,354,890 (19.7)	1088 (24.4)	1,355,978 (19.7)	<0.001
30–59 years	3,728,493 (54.3)	2069 (46.5)	3,730,562 (54.3)	
60–79 years	1,782,492 (26.0)	1296 (29.1)	1,783,788 (26.0)	
Gender				
Men	3,432,214 (50.0)	2526 (56.7)	3,434,740 (50.0)	<0.001
Women	3,433,661 (50.0)	1927 (43.3)	3,435,588 (50.0)	
Birth country				
Sweden	5,428,635 (79.1)	3697 (83.0)	5,432,332 (79.1)	<0.001
Outside Sweden	1,437,240 (20.9)	756(17.0)	1,437,996 (20.9)	
Income				
Low (1st quartile)	1,560,187 (22.7)	1074 (24.1)	1,561,261 (22.7)	0.011
Medium low (2nd quartile)	1,557,806 (22.7)	1035 (23.2)	1,558,841 (22.7)	
Medium high (3rd quartile)	1,754,138 (25.5)	1150 (25.8)	1,755,288 (25.5)	
High (4th quartile)	1,991,898 (29.0)	1194 (26.8)	1,993,092 (29.0)	
Unknown	1846 (0.0)	0 (0.0)	1 846 (0.0)	
Education				
Primary (<9 years)	925,325 (13.5)	751 (16.9)	926,076 (13.5)	<0.001
Secondary (9–12 years)	3,080,678 (44.9)	2157 (48.4)	3,082,835 (44.9)	
Tertiary (>12 years)	2,744,095 (40.0)	1482 (33.3)	2,745,577 (40.0)	
Unknown	115,777 (1.7)	63 (1.4)	115,840 (1.7)	
Occupation				
Healthcare worker	513,127 (7.5)	334 (7.5)	513,461 (7.5)	<0.001
Other essential worker	1,010,631 (14.7)	613 (13.8)	1,011,244 (14.7)	
Not essential worker	4,114,902 (59.9)	2567 (57.6)	4,117,469 (59.9)	
Unemployed	1,227,215 (17.9)	939 (21.1)	1,228,154 (17.9)	
Civil status				
Married	2,879,687 (41.9)	1682 (37.8)	2,881,369 (41.9)	<0.001
Not married	3,984,342 (58.0)	2771 (62.2)	3,987,113 (58.0)	
Unknown	1846 (0.0)	0 (0.0)	1 846 (0.0)	
Family history of type 1 diabetes				
No	6,811,864 (99.2)	4280 (96.1)	6,816,144 (99.2)	<0.001
Yes	54,011 (0.8)	173 (3.9)	54,184 (0.8)	
Cardiovascular diseases				
No	6,772,065 (98.6)	4333 (97.3)	6,776,398 (98.6)	<0.001
Yes	93,810 (1.4)	120 (2.7)	93,930 (1.4)	
Hypertension				
No	6,468,136 (94.2)	3864 (86.8)	6,472,000 (94.2)	<0.001
Yes	397,739 (5.8)	589 (13.2)	398,328 (5.8)	
Chronic lung disease				
No	6,811,022 (99.2)	4392 (98.6)	6,815,414 (99.2)	<0.001
Yes	54,853 (0.8)	61 (1.4)	54,914 (0.8)	
Asthma				
No	6,750,235 (98.3)	4345 (97.6)	6,754,580 (98.3)	<0.001
Yes	115,640 (1.7)	108 (2.4)	115,748 (1.7)	
Chronic kidney disease				
No	6,771,967 (98.6)	4293 (96.4)	6,776,260 (98.6)	<0.001
Yes	93,908 (1.4)	160 (3.6)	94,068 (1.4)	
Autoimmune diseases				
No	6,728,123 (98.0)	4309 (96.8)	6,732,432(98.0)	<0.001
Yes	137,752 (2.0)	144 (3.2)	137,896 (2.0)	
Dementia				
No	6,849,083 (99.8)	4434 (99.6)	6,853,517 (99.8)	0.014
Yes	16,792 (0.2)	19 (0.4)	16,811 (0.2)	
Psychiatric conditions				
No	6,576,163 (95.8)	4237 (95.1)	6,580,400 (95.8)	0.036
Yes	289,712 (4.2)	216 (4.9)	289,928 (4.2)	
Obesity				
No	6,753,029 (98.4)	4359 (97.9)	6,757,388 (98.4)	0.014
Yes	112,846 (1.6)	94 (2.1)	112,940 (1.6)	
Cancer				
No	6,574,437 (95.8)	4181 (93.9)	6,578,618 (95.8)	<0.001
Yes	291,438 (4.2)	272 (6.1)	291,710 (4.2)	
VOC periods at first infection				
No infection	5,047,743 (73.5)	3870 (86.9)	5,051,613 (73.5)	<0.001
PreAlpha (1 January 2020 to 31 January 2021)	456,797 (6.7)	171 (3.8)	456,968 (6.7)	
Alpha (1 February 2021 to 30 Jun 2021)	362,032 (5.3)	144 (3.2)	362,176 (5.3)	
Delta (1 July 2021 to 31 December 2021)	173,474 (2.5)	47 (1.1)	173,521 (2.5)	
Omicron (from 1 January 2022)	825,829 (12.0)	221 (5.0)	826,050 (12.0)	
Vaccination				
Unvaccinated	1,025,479 (14.9)	2431 (54.6)	1,027,910 (15.0)	<0.001
1–2 doses	1,300,709 (18.9)	865 (19.4)	1,301,574 (18.9)	
3 and more doses	4,539,687 (66.1)	1157 (26.0)	4,540,844 (66.1)	

Values are presented as *n* (percentages)

*p* values are obtained from *χ*^2^ test

### Statistical method

All analyses were done for children and adults separately, using STATA (version 18.0; StataCorp) and R.

#### Data preparation for analysis

Two datasets were prepared for children and adults, respectively: one for the infection analysis and one for the vaccination analysis.

For the dataset for infection analysis, the follow-up started on 1 January 2020 (or the date of birth for individuals born during follow-up) and ended at the earliest of incident type 1 diabetes diagnosis, 2 years after first SARS-CoV-2 infection, or a censoring event (death, emigration or end of study period). Each individual’s follow-up time was first split by infection status (uninfected vs infected) and, within the infected period, further split into the prespecified post-infection risk windows. Follow-up time was additionally split whenever an individual’s vaccination status changed. For example, if a person received their first vaccine dose on day 50 after infection, their person-time within the 31–180-day post-infection window was further divided into 31–49 days (unvaccinated), and 50–180 days (vaccinated, 1–2 doses).

For the dataset for vaccination analysis, the follow-up started on 27 December 2020 when first vaccination was available in Sweden (or date of birth) and ended at the earliest of incident type 1 diabetes diagnosis, 2 years without any new booster vaccination after the receipt of the latest dose (up to dose 3), or a censoring event (receipt of a fourth COVID-19 vaccine dose, death, emigration, end of study period). Each individual’s follow-up time was first split by vaccination status (unvaccinated vs vaccinated for children; unvaccinated, dose 1, dose 2, dose 3 for adults) and then further split into the prespecified post-vaccination risk windows within each dose category.

#### COX regression

We used Cox proportional hazard regression models with time-varying exposure, using calendar time as the underlying timescale. This approach allows risk sets to be assessed sequentially in the model each day, thus by design controlling for calendar time-related factors such as infection pressure or pandemic restrictions. We estimated hazard ratio (HR) and 95% confidence interval (CI) for the overall infected period vs uninfected period, as well as for each risk window after infection vs uninfected period. Two models were assessed, one crude (unadjusted) model and one full model with all covariates listed. Gender was included in the adjusted model to account for the potential impact, however, gender stratified analysis was not considered.

For the study aim on vaccination and type 1 diabetes, a similar approach was applied, and HRs with 95% CI were reported for overall comparison between vaccinated period and unvaccinated period, as well as for each risk window after each dose.

To investigate whether vaccination can alter the risk of type 1 diabetes related to infection, we added interaction terms (vaccination status × infection; or vaccination status × risk window post-infection) into the full model. HR with 95% CI for unvaccinated and vaccinated periods (children) and for unvaccinated, 1–2 doses and ≥3 doses (adults) were reported together with *p* values for interaction.

#### Sensitivity analyses

We performed a sensitivity analysis for the infection analysis by truncating the study period on 9 February 2022. This date coincides with the cessation of large-scale COVID-19 testing, after which mostly only severe cases of SARS-CoV-2 infection, where healthcare visits were required, were tested and captured in SmiNet if positive. This shift introduced a potential misclassification of infection (mild infection could not be captured and was thus classified as no infection). Additionally, as vaccination was recommended to children from 12 years of age in Sweden, we performed another sensitivity analysis for vaccination analysis among children by excluding children 0–11 years old at the study start and newborns during the study period.

## Results

The study included 2,650,492 children (<18 years at the study start and newborns during the study period), of whom 3813 developed incident type 1 diabetes during the study period, and 6,870,328 adults (18–79 years at the study start), of whom 4453 developed incident type 1 diabetes. Among children who developed type 1 diabetes, boys were over-represented (57.3%, vs 51.5% in the general population). These children were also more likely to have been born in Sweden, to have Sweden-born parents, and to have a family history of type 1 diabetes (10.0% vs 1.4%) (Table 1). Among adults with incident type 1 diabetes, ages 18–29 and 60–79 were more common, and the majority were male (56.7%). Several comorbidities, including hypertension, cardiovascular disease, chronic kidney disease and cancer, were more prevalent in adults who developed type 1 diabetes compared with the general population (Table 2).

**Fig. 1:**
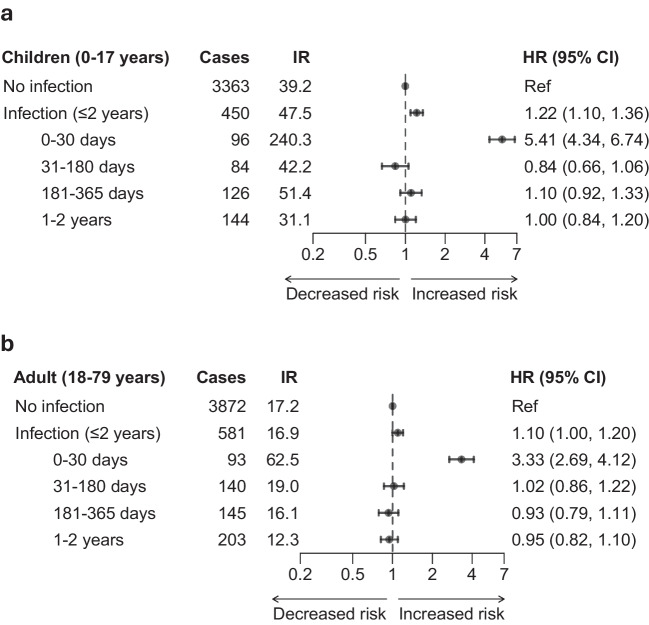
Number of cases, IR (per 100,000 person-years), as well as HR (dots) with 95% CI (lines) of type 1 diabetes within 2 years following SARS-CoV-2 infection, and within shorter risk windows, among Swedish children (**a**) and adults (**b**), respectively. HR and 95% CI were obtained from the fully adjusted model. The detailed data are presented in ESM Table 2 (full model)

Crude IRs of type 1 diabetes were much higher among children than adults (Fig. 1). The overall risk of incident type 1 diabetes within the 2-year window after SARS-CoV-2 infection was elevated for both children (HR 1.22; 95% CI 1.10, 1.36) and adults (1.10; 1.00, 1.20) in the fully adjusted model, with the highest risks observed in the 0–30-day window post-infection (5.41 for children, 3.33 for adults). Beyond 30 days, risk estimates decreased substantially and were no longer associated with infection (Fig. 1). No major difference between the crude and full models was noted, indicating marginal confounding effects by the comprehensive covariate set (ESM Table 2). The sensitivity analysis by truncating the study period on 9 February 2022 to deal with the potential misclassification of SARS-CoV-2 infection showed similar patterns of post-infection type 1 diabetes risk across the risk windows (ESM Table 3).

**Fig. 2:**
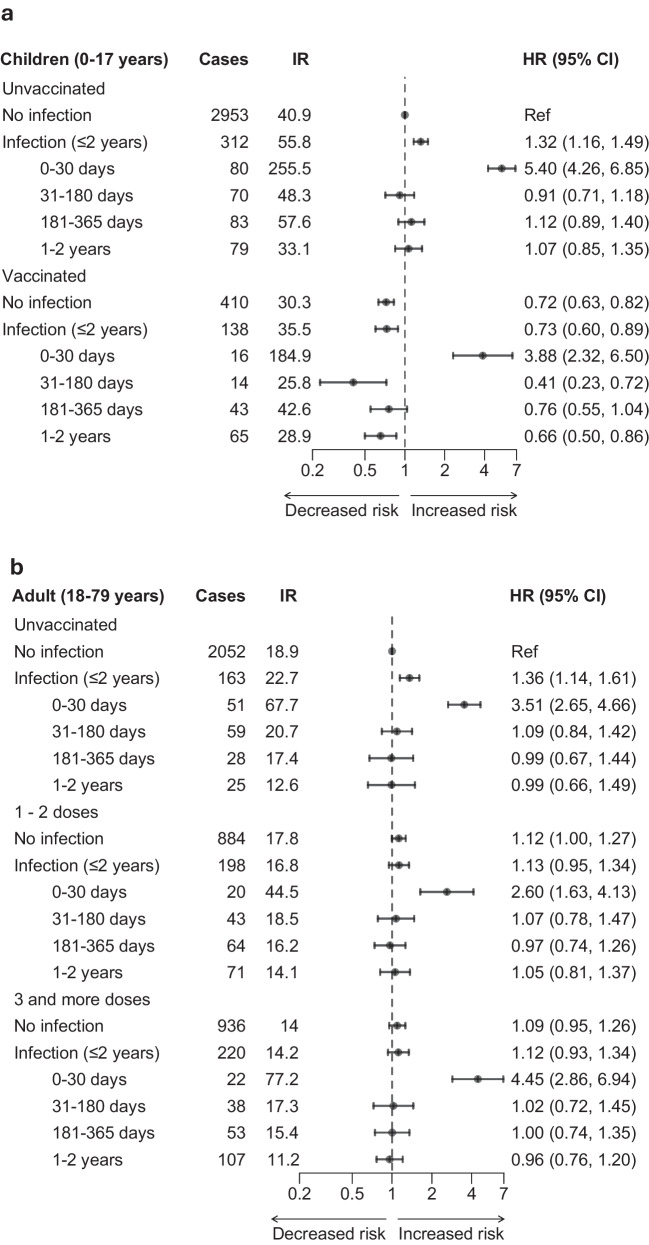
Number of cases, IR (per 100,000 person-years), as well as HR (dots) and 95% CI (lines) of type 1 diabetes within 2 years following SARS-CoV-2 infection, and within shorter risk windows, among Swedish children (**a**) and adults (**b**), stratified by vaccination status. HR and 95% CI were obtained from the fully adjusted model with interaction term. Unvaccinated and uninfected period was used as the only reference group. The detailed data are presented in ESM Table 4 (full model)

Overall, vaccination status did not show any clear modifying effect on the association between SARS-CoV-2 infection and the risk of type 1 diabetes in either children or adults (interaction *p* value >0.5 for both), although some changes in the estimates emerged. Compared with uninfected and unvaccinated children, the 0–30-day post-infection risk was elevated in both unvaccinated (HR 5.40; 4.26, 6.85) and vaccinated (3.88; 2.32, 6.50) children. From the 31–180 days risk window onward, vaccinated children consistently showed HRs below 1, while the unvaccinated children retained HRs around 1 (Fig. 2a, ESM Table 4). In the sensitivity analysis restricted to children aged 12–17 years, the decreased HRs of type 1 diabetes following SARS-CoV-2 infection in vaccinated children were not observed (ESM Fig. 1, ESM Table 5). In adults, the effect modification by vaccination was even weaker and less evident. Among the unvaccinated adults, SARS-CoV-2 infection was associated with 1.36 times higher risk of type 1 diabetes compared to uninfected adults. This increased risk was slightly attenuated in vaccinated adults (HR 1.12 for 1–2 doses, and 1.09 for ≥3 doses), with similar patterns of risks observed in both vaccinated and unvaccinated adults. Elevated risks were only apparent in the 0–30 day risk window post-infection (Fig. 2b, ESM Table 4).

**Fig. 3:**
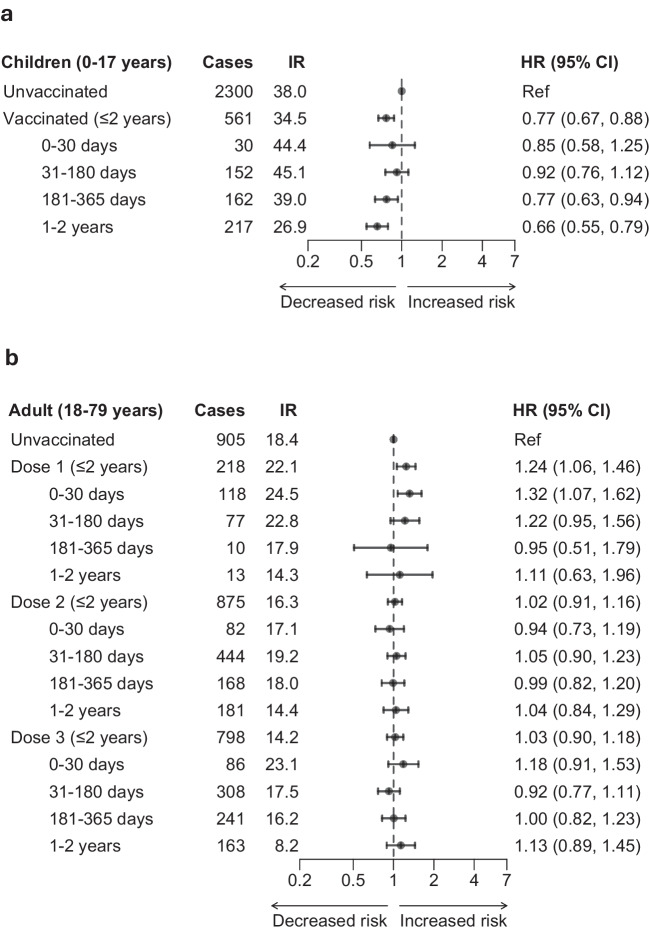
Number of cases, IR (per 100,000 person-years), as well as HR (dots) and 95% CI (lines) of type 1 diabetes within 2 years following COVID-19 vaccination, and within shorter risk windows, among Swedish children (**a**) and adults (**b**), respectively. HR and 95% CI were obtained from the fully adjusted model. The detailed data are presented in ESM Table 5 (full model)

When analysing vaccination as the primary exposure, children and adults showed slightly different patterns (Fig. 3, ESM Table 6). Among children, vaccination was associated with an overall lower type 1 diabetes risk compared to those who remained unvaccinated (HR 0.77; 95% CI 0.67, 0.88), with estimates below 1 across all post-vaccination windows. The negative association was more pronounced in later windows (i.e. beyond 180 days). However, in the sensitivity analysis restricted to children aged 12–17 years, this pattern disappeared (ESM Fig. 2, ESM Table 7). Among adults, an excess risk estimate was only seen for dose 1 within the 0–30 days post-vaccination window (1.32; 1.07, 1.62).

## Discussion

In this nationwide register-based cohort of more than 9.5 million individuals, we observed an apparent increase in type 1 diabetes diagnoses in the immediate period after SARS-CoV-2 infection, with an association concentrated to the first 30 days after infection in both children and adults. Importantly, beyond 30 days after infection, the association disappeared. Vaccination did not modify infection-associated risk overall, and when vaccination itself was treated as the exposure there was no consistent signal of association to incident type 1 diabetes: adults showed only a discretely elevated HR limited to the 0–30 day window after dose 1. Although the analysis for children showed somewhat lower hazards overall, these disappeared in the sensitivity analysis restricted to adolescents (12–17 years). Overall, we interpret this short-term elevation of type 1 diabetes diagnosis after infection or vaccination primarily as detection bias rather than evidence that infection or vaccination have caused new-onset type 1 diabetes. Such findings can be generalised to all genders in the population.

The time-restricted elevated risk within 0–30-day post-infection in our study aligns with several population-based studies that also observed clustering of type 1 diabetes diagnoses near the time of infection but little to no association after a 1 month lag. In a Scottish national cohort of individuals aged <35 years, the rate ratio for incident type 1 diabetes was 2.62 in the 0–30 days after a first positive SARS-CoV-2 test, but 0.86 for infections >30 days earlier, suggesting the observed increase was unlikely to be caused by the virus itself and may partly reflect earlier detection around illness or testing [9]. Similarly, a Danish nationwide analysis reported no increase in first-time type 1 diabetes diagnoses more than 30 days after infection in children [10]. A recent Swedish study also supports the view that any association is largely short term and may reflect diagnostic acceleration rather than new disease. In a population-based matched cohort study among individuals under 30 years in Sweden, covering 2007–2023, the overall adjusted HR for developing type 1 diabetes after SARS-CoV-2 infection was close to null, despite a higher risk of diagnosis within the first 28 days after infection among children aged 5–10 years. Taken together, these previous findings are consistent with our central interpretation that acute infection increases the probability that individuals already near clinical onset are diagnosed, rather than meaningfully increasing the long-term risk of type 1 diabetes in the general population [6].

There are several possible explanations for how SARS-CoV-2 infection might inflate incident type 1 diabetes diagnoses in the short term. Respiratory infections often lead to clinical encounters where blood tests are more likely to be performed, increasing diagnostic intensity, and therefore pre-symptomatic diabetes could be detected ‘by chance’. More importantly, the infection would increase insulin resistance [32], and impose acute metabolic stress, acting as a ‘diagnostic accelerator’ for individuals with advanced, yet undiagnosed beta cell failure [33, 34]. In this context, SARS-CoV-2 infection may shift the timing of clinical diagnosis forward, rather than induce de novo autoimmunity. Thus, infection may precipitate acute metabolic decompensation, prompting earlier medical presentation and subsequent diagnosis of type 1 diabetes. Reverse causation cannot be fully excluded either, as individuals with emerging but undiagnosed type 1 diabetes may already have hyperglycaemia or metabolic disturbances that might increase susceptibility to SARS-CoV-2 infection or probability of healthcare contact. However, no strong evidence supports that diabetes, especially type 1 diabetes, would increase the risk of contracting SARS-CoV-2 infection, although diabetes is strongly associated with worse outcomes once infected [35].

We found no clear interaction between pre-infection vaccination status and the association between infection and incident type 1 diabetes, suggesting that vaccination did not significantly alter the infection and type 1 diabetes association. If the post-infection sharp increased risk reflected a true causal diabetogenic effect of SARS-CoV-2, one might hypothesise a stronger attenuation among vaccinated individuals due to lower infection severity. However, the absence of a clear modifying effect is therefore consistent with the notion that the short-term association is largely driven by healthcare contact patterns rather than infection itself.

There is limited evidence supporting an association between COVID-19 vaccination and new-onset type 1 diabetes. A Nordic study with register data from Norway and Sweden showed no indication of any consistent large effect of COVID-19 vaccination on type 1 diabetes in children and young adults [36]. Ecological study in Germany also found no association between childhood COVID-19 vaccination rates and the subsequent incidence of type 1 diabetes over the next 12 months [17]. In our study, the increased risk during the first 30 days after first dose but not after subsequent doses in adults strongly indicates the type of temporal clustering expected from increased medical contact around vaccination and increased symptom awareness. On the other hand, the apparently lower risk of type 1 diabetes after vaccination observed in the full children cohort disappeared when we restricted the analysis to adolescents. This pattern likely reflects selection bias in the full children cohort, since in Sweden, COVID-19 vaccination was primarily recommended for children aged ≥12 years; therefore, vaccinated children younger than 12 years were not representative of the general child population, but were more likely to belong to vulnerable groups (e.g. with pre-existing health conditions) who were vaccinated earlier. As a result, the vaccinated group in the full children cohort may have differed systematically from unvaccinated children, which could create the appearance of a lower post-vaccination risk that does not persist in the age-restricted analysis. Therefore, our findings support the conclusion that there is no association between COVID-19 vaccination and new-onset type 1 diabetes in either children or adults.

Our study benefits from a very large population-based design, with the ability to analyse children and adults separately in the same population, apply time-varying definitions for infection-related windows, and evaluate vaccination both as an effect modifier and as an exposure. Nevertheless, several potential limitations warrant consideration. Outcome misclassification may occur, particularly among adults where clinical diagnosis can conflate classical type 1 diabetes, latent autoimmune diabetes in adults (LADA), and insulin-requiring type 2 diabetes. Exposure misclassification is also possible, as registered infection cases depend on evolving testing practices and healthcare-seeking behaviour. Undetected infections would likely attenuate true associations if they were non-differential. However, differential misclassification may occur if individuals experiencing symptoms common to both infection and diabetes (e.g. fatigue) were more likely to be tested. To assess the potential impact of this bias, we compared our main analysis (which included the period after large-scale testing ceased) with a sensitivity analysis restricted to the period before 9 February 2022, when testing was widespread and in principle mandatory. The similarity of results across these analyses suggests that infection misclassification is likely to be marginal. Finally, certain comorbidities, such as hypertension, are primarily managed in primary healthcare. Because our study lacked access to national primary healthcare data, these relatively mild comorbidities may be under-reported or misclassified as covariates in our adjusted models.

### Conclusion

We observed short-term elevations in incident type 1 diabetes diagnoses in the first 30 days after infection and after first vaccination, but the elevation did not persist. Such short-term signals most plausibly reflect when type 1 diabetes diagnoses are made, not that they are caused by these exposures.

## Supplementary Information

Below is the link to the electronic supplementary material.

ESM (PDF 587 KB)

## Data Availability

This study used pseudonymised individual-level data from Swedish healthcare registers that are not publicly available according to Swedish legislation. The data can be obtained from the respective Swedish data holders on the basis of ethics approval for the research in question, subject to relevant legislation, processes and data protection.

## Abbreviations

CI: Confidence interval
HR: Hazard ratio
IR: Incidence rate
NDR: National Diabetes Register
NPR: National Patient Register
VOC: Variant of concern

## Authors’ relationships and activities

FN owns some AstraZeneca shares. FN, HL and AS report participation in research projects funded by Bayer and AstraZeneca (regulator-mandated phase IV study; investigator-initiated study), with funds paid to the University of Gothenburg where they are employed (no personal fees) and with no relation to the work reported here. KE-O has been a consultant for Novo Nordisk, Sanofi, Eli Lilly and Abbott Diabetes Care and a lecturer at Sanofi and Abbott Diabetes Care. The remaining authors declare that there are no relationships or activities that might bias, or be perceived to bias, their work.
