# Supplementary material for: SARS-CoV-2 infection and COVID-19 vaccination and the risk for new-onset type 1 diabetes: a register-based population study in Sweden
Source: Diabetologia. 2026 Jun 6;69(9):2498–511. doi: 10.1007/s00125-026-06767-6 (PMC13424464; doi:10.1007/s00125-026-06767-6)
Supplement: Supplementary file 1 — ESM (PDF 587 KB) [file 125_2026_6767_MOESM1_ESM.pdf]

SARS-CoV-2 infection and COVID-19 vaccination and the risk for new onset type 1 diabetes: a register-based population study in Sweden

Huiqi Li (1), Lisa Morris (2,3), Maria Bygdell (3), Ailiana Santosa (1), Elin Allansson Kjölhede (4, 5), Katarina Eeg-Olofsson (4,5, 6), Fredrik Nyberg (1), Yiyi Xu(1*)

Affiliations:

(1) School of Public Health and Community Medicine, Institute of Medicine, Sahlgrenska Academy, University of Gothenburg, Gothenburg, Sweden

(2) Unit of Clinical Pharmacology, Department of Pharmaceuticals, Sahlgrenska University Hospital, Region Västra Götaland, Gothenburg, Sweden.

(3) Department of Internal Medicine and Clinical Nutrition, Institute of Medicine, Sahlgrenska Academy, University of Gothenburg, Gothenburg, Sweden.

(4) Department of Medicine, Sahlgrenska University Hospital, Gothenburg, Sweden

(5) Department of Molecular and Clinical Medicine, Sahlgrenska Academy, Gothenburg, Sweden.

(6) Centre of Registers Västra Götaland, Gothenburg, Sweden.

* Corresponding author, yiyi.xu@amm.gu.se

Contents

Supplemental Tables 3

ESM Table 1. International Classification of Diseases, 10th revision (ICD-10) codes for baseline comorbidities........ 3

ESM Table 2. Incidence rates, as well as hazard ratios (HR) with 95% confidence interval (CI), for incident type 1 diabetes *within 2 years following **infection** and within shorter risk windows*, among children and adults separately. 4

ESM Table 3. Incidence rates, as well as hazard ratios (HR) with 95% confidence interval (CI), for incident type 1 diabetes *within 2 years following **infection** and within shorter risk windows*, among children and adults separately; **using a shorter study period from 1 January 2020 to 9 February 2022** when large scale COVID-19 testing ended. 6

ESM Table 4. Incidence rates, as well as hazard ratios (HR) with 95% confidence interval (CI) for incident type 1 diabetes *within 2 years following **infection** and within shorter risk windows*, **separately by vaccine status**, among children and adults separately 8

ESM Table 5. Incidence rates, as well as hazard ratios (HR) with 95% confidence interval (CI) for incident type 1 diabetes *within 2 years following **infection** and within shorter risk windows,* **separately by vaccine status, among children aged 12-17 years** 11

ESM Table 6. Incidence rates, as well as hazard ratios (HR) with 95% confidence interval (CI) for incident type 1 diabetes *within 2 years following **vaccination** by each dose and risk windows*, among children and adults separately........ 12

ESM Table 7. Incidence rates, as well as hazard ratios (HR) with 95% confidence interval (CI) for incident type 1 diabetes *within 2 years following **vaccination** by each dose and risk windows*, **among children 12-17 years.** 14

Supplemental Figures........ 15

ESM Figure 1. Count of cases, incidence rate (IR), as well as hazard ratios (HR, dots) and 95% confidence intervals (CI, lines) of type 1 diabetes *within 2 years following **infection**, and within shorter risk windows*, **among children aged 12-17 years** who were recommended to get COVID-19 vaccination, **stratified by vaccination status**. 15

ESM Figure 2. Count of cases, incidence rate (IR), as well as hazard ratios (HR, dots) and 95% confidence intervals (CI, lines) of type 1 diabetes *within 2 years following **vaccination**, and within shorter risk windows,* **among children aged 12-17 years** who were recommended to get COVID-19 vaccination, respectively. 16

Supplemental Tables

ESM Table 1. International Classification of Diseases, 10th revision (ICD-10) codes for baseline comorbidities. All comorbidities were defined using primary or secondary diagnoses from in- or outpatient specialist care obtained from the National Patient Register (NPR) during the five years before 1 Jan 2020.

**Prior comorbidities**	**ICD-10**
Cardiovascular diseases (for adult analysis only)	I05-I09, I20-I51, R001, R011, Q20-Q28
Hypertension (for adult analysis only)	I10-I15
Chronic lung diseases (for adult analysis only)	J430 J431 J432 J438 J439 J448 J449 J840 J841 J848 J849 J961 J969 E840 E841 E848 E849
Asthma	J45
Chronic kidney diseases (for adult analysis only)	N18-N19
Autoimmune diseases (for adult analysis only)	M05-M14
Dementia (for adult analysis only)	F00-F03
Psychiatric conditions (for adult analysis only)	F20-F29, F30-F39
Down’s syndrome (for children analysis only)	Q90
Obesity	E66
Cancer (for adult analysis only)	C00-C97

ESM Table 2. Incidence rates, as well as hazard ratios (HR) with 95% confidence interval (CI), for incident type 1 diabetes *within 2 years following **infection** and within shorter risk windows*, among children and adults separately.

Risk windows	Person-years	Cases	Incidence rate	Crude model			Full model *		
			(Per 100 000 person-years)	HR	low CI	up CI	HR	low CI	up CI
Among children									
Uninfected	8577055	3363	39.2	Ref			Ref		
Infected. up to 2 years	947621	450	47.5	1.24	1.12	1.37	1.22	1.10	1.36
0-30d	39958	96	240.3	5.67	4.56	7.04	5.41	4.34	6.74
31-180d	199237	84	42.2	0.92	0.74	1.15	0.84	0.66	1.06
181d-one year	245068	126	51.4	1.10	0.91	1.32	1.10	0.92	1.33
1-2 years	463358	144	31.1	0.99	0.83	1.17	1.00	0.84	1.20
Among adults									
Uninfected	22490793	3872	17.2	Ref			Ref		
Infected. up to 2 years	3441551	581	16.9	1.04	0.95	1.14	1.10	1.00	1.20
0-30d	148819	93	62.5	3.20	2.58	3.96	3.33	2.69	4.12
31-180d	736460	140	19.0	0.98	0.82	1.16	1.02	0.86	1.22
181d-one year	900778	145	16.1	0.89	0.75	1.05	0.93	0.79	1.11
1-2 years	1655494	203	12.3	0.90	0.77	1.04	0.95	0.82	1.10

* Full model for children infection analysis: Age (0-11y, 12-17y), gender (boys, girls), birth country (Sweden, outside Sweden), parents' birth country (both from Sweden, either from Sweden, none from Sweden, unknown), Family disposable income (low, medium low, medium high, high, unknown), mother's education (primary, secondary, tertiary, unknown), father's education (primary, secondary, teritary, unknown), mother's occupation (healthcare worker, other essential worker, non-essential worker, unemployed), father's

occupation (healthcare work, other essential worker, non-essential worker, unemployed), family history of T1D (yes, no), asthma (yes, no), downs syndrome (yes, no), obesity (yes, no), vaccination status (time varying: unvaccinated, vaccinated).

Full model for adult infection analysis: Age (18-29y, 30-59y, 60-79y), gender (men, women), birth country (Sweden, outside Sweden), income (low, medium low, medium high, high, unknown), education (primary, secondary, tertiary, unknown), occupation (healthcare worker, other essential worker, non-essential worker, unemployed), civil status (married, not married, unknown), family history of T1D (yes, no), CVD (yes, no), hypertension (yes, no), chronic lung disease (yes, no), asthma (yes, no), chronic kidney disease (yes, no), autoimmune diseases (yes, no), dementia (yes, no), psychiatric conditions (yes, no), obesity (yes, no), cancer (yes, no), vaccination status (time varying: unvaccinated, max 2 doses, ≥3 doses).

ESM Table 3. Incidence rates, as well as hazard ratios (HR) with 95% confidence interval (CI), for incident type 1 diabetes *within 2 years following* ***infection*** *and within shorter risk windows*, among children and adults separately; **using a shorter study period from 1 January 2020 to 9 February 2022** when large scale COVID-19 testing ended.

Risk windows	Person-years	Cases	Incidence rate	Full model *		
			(Per 100 000 person-years)	HR	low CI	up CI
Among children						
Uninfected	4718478	2132	45.2	Ref		
Infected. up to 2 years	167108	118	70.6	1.46	1.20	1.78
0-30d	32480	62	190.9	4.15	3.17	5.45
31-180d	78590	34	43.3	0.87	0.61	1.24
181d-one year	51398	22	42.8	0.91	0.59	1.39
1-2 years	4640	0				
Among adults						
Uninfected	13326717	2627	19.7	Ref		
Infected. up to 2 years	751169	142	18.9	0.99	0.84	1.18
0-30d	117479	62	52.8	2.75	2.12	3.58
31-180d	326620	53	16.2	0.84	0.63	1.10
181d-one year	289173	27	9.3	0.50	0.34	0.74
1-2 years	17897	0				

* Full model for children infection analysis: Age (0-11y, 12-17y), gender (boys, girls), birth country (Sweden, outside Sweden), parents' birth country (both from Sweden, either from Sweden, none from Sweden, unknown), Family disposable income (low, medium low,

medium high, high, unknown), mother's education (primary, secondary, tertiary, unknown), father's education (primary, secondary, teritary, unknown), mother's occupation (healthcare worker, other essential worker, non-essential worker, unemployed), father's occupation (healthcare work, other essential worker, non-essential worker, unemployed), family history of T1D (yes, no), asthma (yes, no), downs syndrome (yes, no), obesity (yes, no), vaccination status (time varying: unvaccinated, vaccinated).

Full model for adult infection analysis: Age (18-29y, 30-59y, 60-79y), gender (men, women), birth country (Sweden, outside Sweden), income (low, medium low, medium high, high, unknown), education (primary, secondary, tertiary, unknown), occupation (healthcare worker, other essential worker, non-essential worker, unemployed), civil status (married, not married, unknown), family history of T1D (yes, no), CVD (yes, no), hypertension (yes, no), chronic lung disease (yes, no), asthma (yes, no), chronic kidney disease (yes, no), autoimmune diseases (yes, no), dementia (yes, no), psychiatric conditions (yes, no), obesity (yes, no), cancer (yes, no), vaccination status (time varying: unvaccinated, max 2 doses, ≥3 doses).

ESM Table 4. Incidence rates, as well as hazard ratios (HR) with 95% confidence interval (CI) for incident type 1 diabetes *within 2 years following **infection** and within shorter risk windows*, **separately by vaccine status**, among children and adults separately

Risk window	Person-years	Cases	Incidence rate	Full model*		
			(Per 100 000 person-years)	HR	low CI	up CI
Among children						
**Unvaccinated period**						
Uninfected	7223516	2953	40.9	Ref		
Infected, up to 2 years	558897	312	55.8	1.32	1.16	1.49
Infected, 0-30d	31305	80	255.5	5.40	4.26	6.85
Infected, 31-180d	144911	70	48.3	0.91	0.71	1.18
Infected, 181-365d	144114	83	57.6	1.12	0.89	1.40
Infected, 1-2 years	238568	79	33.1	1.07	0.85	1.35
**Vaccinated period**						
Uninfected	1353539	410	30.3	0.72	0.63	0.82
Infected, up to 2 years	388724	138	35.5	0.73	0.60	0.89
Infected, 0-30d	8653	16	184.9	3.88	2.32	6.50
Infected, 31-180d	54326	14	25.8	0.41	0.23	0.72
Infected, 181-365d	100955	43	42.6	0.76	0.55	1.04
Infected, 1-2 years	224790	65	28.9	0.66	0.50	0.86
Among adults						

**Unvaccinated period**						
Uninfected	10838919	2052	18.9	ref		
Infected, up to 2 years	718798	163	22.7	1.36	1.14	1.61
Infected, 0-30d	75377	51	67.7	3.51	2.65	4.66
Infected, 31-180d	284382	59	20.7	1.09	0.84	1.42
Infected, 181-365d	161134	28	17.4	0.99	0.67	1.44
Infected, 1-2 years	197905	25	12.6	0.99	0.66	1.49
**Max 2 doses**						
Uninfected	4952670	884	17.8	1.12	1.00	1.27
Infected, up to 2 years	1177688	198	16.8	1.13	0.95	1.34
Infected, 0-30d	44962	20	44.5	2.60	1.63	4.13
Infected, 31-180d	232888	43	18.5	1.07	0.78	1.47
Infected, 181-365d	396181	64	16.2	0.97	0.74	1.26
Infected, 1-2 years	503657	71	14.1	1.05	0.81	1.37
**3 and more doses**						
Uninfected	6699204	936	14.0	1.09	0.95	1.26
Infected, up to 2 years	1545065	220	14.2	1.12	0.93	1.34
Infected, 0-30d	28480	22	77.2	4.45	2.86	6.94
Infected, 31-180d	219190	38	17.3	1.02	0.72	1.45
Infected, 181-365d	343463	53	15.4	1.00	0.74	1.35
Infected, 1-2 years	953932	107	11.2	0.96	0.76	1.20

* Full model for children infection analysis: Age (0-11y, 12-17y), gender (boys, girls), birth country (Sweden, outside Sweden), parents' birth country (both from Sweden, either from Sweden, none from Sweden, unknown), Family disposable income (low, medium low, medium high, high, unknown), mother's education (primary, secondary, tertiary, unknown), father's education (primary, secondary, teritary, unknown), mother's occupation (healthcare worker, other essential worker, non-essential worker, unemployed), father's occupation (healthcare work, other essential worker, non-essential worker, unemployed), family history of T1D (yes, no), asthma (yes, no), downs syndrome (yes, no), obesity (yes, no).

Full model for adult infection analysis: Age (18-29y, 30-59y, 60-79y), gender (men, women), birth country (Sweden, outside Sweden), income (low, medium low, medium high, high, unknown), education (primary, secondary, tertiary, unknown), occupation (healthcare worker, other essential worker, non-essential worker, unemployed), civil status (married, not married, unknown), family history of T1D (yes, no), CVD (yes, no), hypertension (yes, no), chronic lung disease (yes, no), asthma (yes, no), chronic kidney disease (yes, no), autoimmune diseases (yes, no), dementia (yes, no), psychiatric conditions (yes, no), obesity (yes, no), cancer (yes, no).

ESM Table 5. Incidence rates, as well as hazard ratios (HR) with 95% confidence interval (CI) for incident type 1 diabetes *within 2 years following **infection** and within shorter risk windows*, **separately by vaccine status, among children aged 12-17 years**

Risk window	Person-years	Cases	Incidence rate	Full model*		
			(Per 100 000 person-years)	HR	low CI	up CI
Unvaccinated period						
Uninfected	1527134	596	39.0	Ref		
Infected, up to 2 years	124017	65	52.4	1.61	1.21	2.13
Infected, 0-30d	11308	25	221.1	5.58	3.66	8.48
Infected, 31-180d	47515	23	48.4	1.16	0.74	1.81
Infected, 181-365d	30707	13	42.3	1.19	0.66	2.14
Infected, 1-2 years	34487	4	11.6	0.60	0.22	1.65
Vaccinated period						
Uninfected	1067698	282	26.4	1.11	0.87	1.42
Infected, up to 2 years	314680	95	30.2	1.13	0.84	1.54
Infected, 0-30d	7067	10	141.5	6.55	3.20	13.38
Infected, 31-180d	44252	10	22.6	0.53	0.25	1.10
Infected, 181-365d	82274	31	37.7	1.14	0.75	1.73
Infected, 1-2 years	181087	44	24.3	0.60	0.22	1.65

ESM Table 6. Incidence rates, as well as hazard ratios (HR) with 95% confidence interval (CI) for incident type 1 diabetes *within 2 years following **vaccination** by each dose and risk windows*, among children and adults separately

Risk window	Person-years	Cases	Incidence rate	Crude model			Full model *		
			(Per 100 000 person-years)	HR	low CI	up CI	HR	low CI	up CI
Among children									
Unvaccinated	6047085	2300	38.0	Ref			Ref		
vaccinated, up to 2 years	1626764	561	34.5	0.87	0.79	0.96	0.77	0.67	0.88
0-30d	67520	30	44.4	0.97	0.67	1.41	0.85	0.58	1.25
31-180d	337360	152	45.1	1.02	0.86	1.21	0.92	0.76	1.12
181d-one year	415541	162	39.0	0.86	0.73	1.02	0.77	0.63	0.94
1-2 years	806343	217	26.9	0.78	0.67	0.90	0.66	0.55	0.79
Among adult									
Unvaccinated	4917652	905	18.4	Ref			Ref		
Dose 1, up to 2 years	986386	218	22.1	1.27	1.08	1.49	1.24	1.06	1.46
0-30d	481376	118	24.5	1.33	1.08	1.63	1.32	1.07	1.62
31-180d	337913	77	22.8	1.25	0.98	1.60	1.22	0.95	1.56
181d-one year	55898	10	17.9	1.02	0.54	1.92	0.95	0.51	1.79
1-2 years	90891	13	14.3	1.19	0.67	2.08	1.11	0.63	1.96
Dose 2, up to 2 years	5354045	875	16.3	1.02	0.91	1.15	1.02	0.91	1.16
0-30d	479686	82	17.1	0.95	0.75	1.22	0.94	0.73	1.19

31-180d	2313040	444	19.2	1.05	0.90	1.23	1.05	0.90	1.23
181d-one year	932113	168	18.0	0.99	0.82	1.21	0.99	0.82	1.20
1-2 years	1253922	181	14.4	1.04	0.84	1.28	1.04	0.84	1.29
Dose 3, up to 2 years	5619493	798	14.2	1.00	0.88	1.14	1.03	0.90	1.18
0-30d	373085	86	23.1	1.19	0.92	1.54	1.18	0.91	1.53
31-180d	1757324	308	17.5	0.94	0.79	1.12	0.92	0.77	1.11
181d-one year	1483961	241	16.2	0.95	0.78	1.16	1.00	0.82	1.23
1-2 years	1987072	163	8.2	1.07	0.84	1.36	1.13	0.89	1.45

* Full model for children vaccination analysis: Age (0-11y, 12-17y), gender (boys, girls), birth country (Sweden, outside Sweden), parents' birth country (both from Sweden, either from Sweden, none from Sweden, unknown), Family disposable income (low, medium low, medium high, high, unknown), mother's education (primary, secondary, tertiary, unknown), father's education (primary, secondary, teritary, unknown), mother's occupation (healthcare worker, other essential worker, non-essential worker, unemployed), father's occupation (healthcare work, other essential worker, non-essential worker, unemployed), family history of T1D (yes, no), asthma (yes, no), downs syndrome (yes, no), obesity (yes, no), VOC period at first infection (no infection, preAlpha, Alpha, Delta, Omicron).

Full model for adult vaccination analysis: Age (18-29y, 30-59y, 60-79y), gender (men, women), birth country (Sweden, outside Sweden), income (low, medium low, medium high, high, unknown), education (primary, secondary, tertiary, unknown), occupation (healthcare worker, other essential worker, non-essential worker, unemployed), civil status (married, not married, unknown), family history of T1D (yes, no), CVD (yes, no), hypertension (yes, no), chronic lung disease (yes, no), asthma (yes, no), chronic kidney disease (yes, no), autoimmune diseases (yes, no), dementia (yes, no), psychiatric conditions (yes, no), obesity (yes, no), cancer (yes, no), VOC period at first infection (no infection, preAlpha, Alpha, Delta, Omicron).

ESM Table 7. Incidence rates, as well as hazard ratios (HR) with 95% confidence interval (CI) for incident type 1 diabetes *within 2 years following* ***vaccination*** *by each dose and risk windows*, **among children 12-17 years.**

Risk window	Person-years	Cases	Incidence rate	Full model *		
			(Per 100 000 person-years)	HR	low CI	up CI
Among children 12-17 years						
Unvaccinated	887840	328	36.9	Ref		
vaccinated, up to 2 years	1273356	384	30.2	1.00	0.80	1.26
0-30d	52643	24	45.6	1.00	0.63	1.58
31-180d	263032	100	38.0	0.98	0.72	1.33
181d-one year	324013	107	33.0	1.12	0.80	1.55
1-2 years	633668	153	24.1	0.96	0.68	1.36

* Full model for children vaccination analysis: Age (0-11y, 12-17y), gender (boys, girls), birth country (Sweden, outside Sweden), parents' birth country (both from Sweden, either from Sweden, none from Sweden, unknown), Family disposable income (low, medium low, medium high, high, unknown), mother's education (primary, secondary, tertiary, unknown), father's education (primary, secondary, teritary, unknown), mother's occupation (healthcare worker, other essential worker, non-essential worker, unemployed), father's occupation (healthcare work, other essential worker, non-essential worker, unemployed), family history of T1D (yes, no), asthma (yes, no), downs syndrome (yes, no), obesity (yes, no), VOC period at first infection (no infection, preAlpha, Alpha, Delta, Omicron).

Supplemental Figures

Children (12-17y)	Cases	IR	HR (95% CI)
Unvaccinated			
No infection	596	39	Ref
Infection (≤ 2 years)	65	52.4	1.61 (1.21 to 2.13)
0-30 days	25	221.1	5.58 (3.66 to 8.48)
31-180 days	23	48.4	1.16 (0.74 to 1.81)
181-365 days	13	42.3	1.19 (0.66 to 2.14)
1-2 years	4	11.6	0.60 (0.22 to 1.65)
Vaccinated			
No infection	282	26.4	1.11 (0.87 to 1.42)
Infection (≤ 2 years)	95	30.2	1.13 (0.84 to 1.54)
0-30 days	10	141.5	6.55 (3.20 to 13.38)
31-180 days	10	22.6	0.53 (0.25 to 1.10)
181-365 days	31	37.7	1.14 (0.75 to 1.73)
1-2 years	44	24.3	0.60 (0.22 to 1.65)

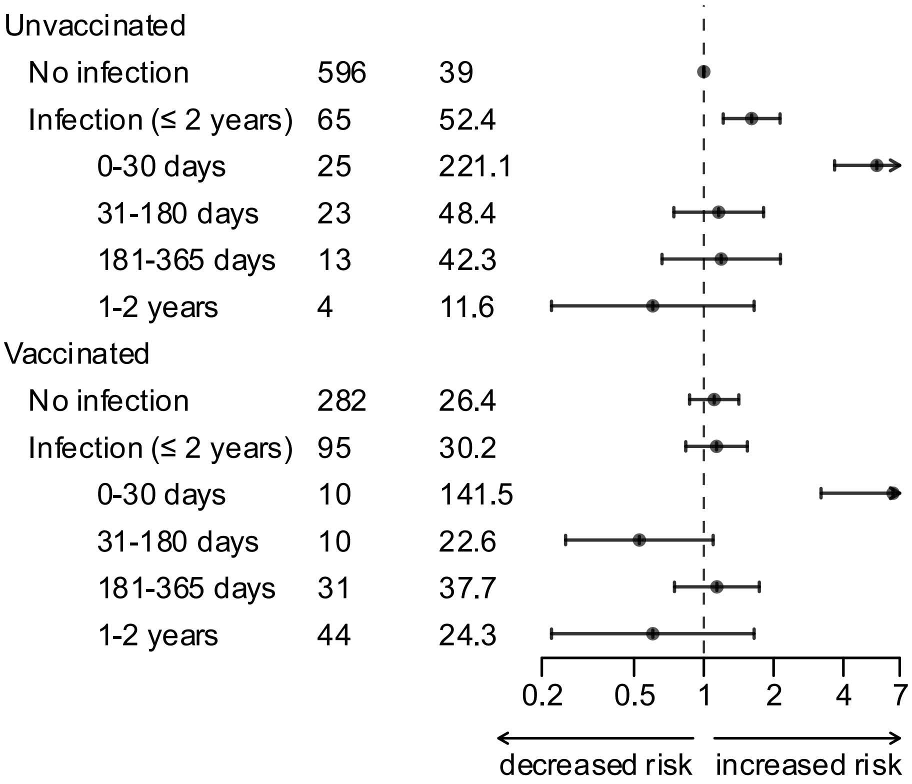

ESM Figure 1. Count of cases, incidence rate (IR), as well as hazard ratios (HR, dots) and 95% confidence intervals (CI, lines) of type 1 diabetes *within 2 years following **infection**, and within shorter risk windows*, **among children aged 12-17 years** who were recommended to get COVID-19 vaccination, **stratified by vaccination status**. The outcome used composite endpoints, including specialist outpatient visits and hospital admissions. HR and 95% CI were obtained from the fully adjusted model with an interaction term of "infection status × vaccination status". The detailed data are presented in ESM Table 5. HR and 95% CI, lines were obtained from the fully adjusted model.

Children (12-17y)	Cases	IR	HR (95% CI)
Unvaccinated	328	36.9	Ref
Vaccinated (≤ 2 years)	384	30.2	1.00 (0.80 to 1.26)
0-30 days	24	45.6	1.00 (0.63 to 1.58)
31-180 days	100	38.0	0.98 (0.72 to 1.33)
181-365 days	107	33.0	1.12 (0.80 to 1.55)
1-2 years	153	24.1	0.96 (0.68 to 1.36)

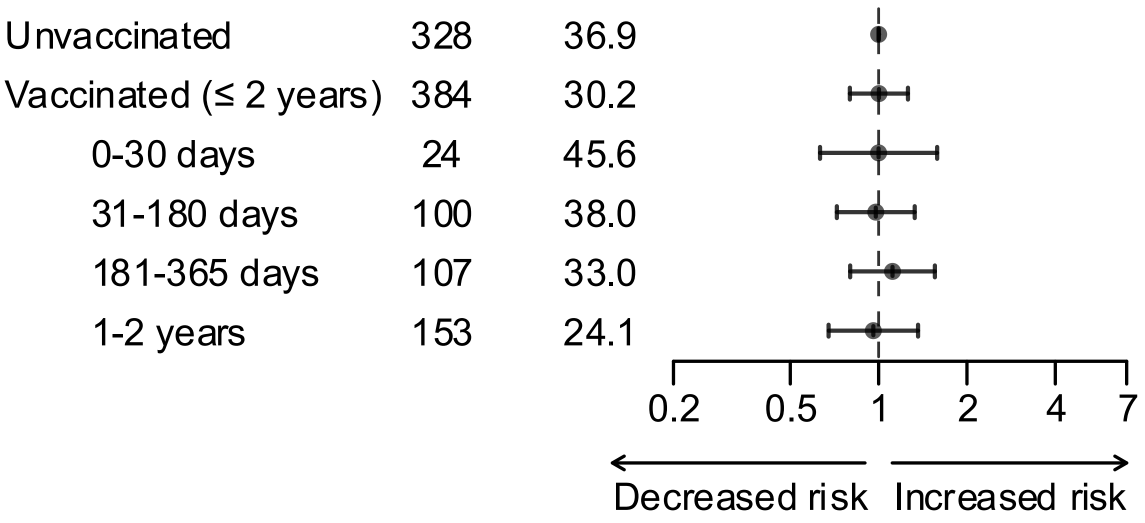

ESM Figure 2. Count of cases, incidence rate (IR), as well as hazard ratios (HR, dots) and 95% confidence intervals (CI, lines) of type 1 diabetes *within 2 years following **vaccination**, and within shorter risk windows,* **among children aged 12-17 years** who were recommended to get COVID-19 vaccination, respectively. The outcome used composite endpoints, including specialist outpatient visits and hospital admissions. The detailed data are presented in ESM Table 7. HR and 95% CI, lines were obtained from the fully adjusted model.
